# An increasing trend of posterior cruciate ligament reconstruction in South Korea: epidemiologic analysis using Korean National Health Insurance System Database

**DOI:** 10.1186/s43019-021-00126-y

**Published:** 2021-12-04

**Authors:** Kyu Sung Chung

**Affiliations:** Department of Orthopedic Surgery and Sports Medical Center and Sports Medical Research Institute, Seoul Paik Hospital, College of Medicine, Inje University, 9, Mareunnae-ro, Jung-gu, Seoul, Korea

**Keywords:** Knee, Posterior cruciate ligament, Reconstruction, Epidemiology, Incidence

## Abstract

**Background:**

The posterior cruciate ligament is crucial for posterior stability of the knee joint, and, as well as anterior cruciate ligament reconstruction, posterior cruciate ligament reconstruction (PCLR) has attracted interest in orthopedic literature. A few studies have investigated epidemiologic data of PCLR in Western countries. However, there has been no report on the epidemiological pattern of PCLR in the Asian population, including South Korea. Therefore, this study investigated the incidence and trends of PCLR in South Korea using the Korean National Health Insurance (NHI) System Database.

**Methods:**

The data was collected by the Korean Health Insurance Review and Assessment Service (HIRA) from 2008 to 2016 in South Korea. Patients with a record of cruciate ligament reconstruction and PCLR were allocated from the database. An analysis of the total number and incidence per 100,000 people/year of PCLR procedures and other epidemiologic parameters was conducted according to sex and age.

**Results:**

The incidence of PCLR procedures rose from 2.3 to 2.6 per 100,000 people (from 1101 to 1299 total cases; 13% increase) between 2008 and 2016: from 3.8 to 4.0 (from 901 to 1000) in males, and from 0.8 to 1.2 (from 200 to 299) in females. PCLR was performed more frequently in males than in females, however, the rate of increase was higher in females than males. The incidence of PCLR over 9  years was highest in patients in their 20s, followed by patients in their 40s and 30s.

**Conclusion:**

The incidence of PCLR procedures increased by 13% over 9 years in South Korea. PCLR was performed approximately three times more in men than in women. The incidence of PCLR was highest in patients in their 20s, followed by those in their 40s. The current study will enhance our understanding of the epidemiology of PCLR.

**Study design:**

Descriptive Epidemiology Study.

## Background

Since the posterior cruciate ligament (PCL) has a high healing potential [1, 2], acute injuries or low-grade instability in the PCL are likely to result in favorable outcomes with conservative treatments [2]. Operative treatments are recommended in patients with grade III posterior instability or combined posterolateral instability that affects subjective instability or physical performance [3]. Long-term evidence shows that conservative treatment leads to progression of osteoarthritis and meniscal tears [4–6]. On the contrary, PCL reconstruction (PCLR) showed favorable outcomes, with a significant reduction of the risks for osteoarthritis, meniscal tears, and cartilage lesions [7, 8]. Thus, interest in PCLR has increased. The average age of the treated patients from PCL injuries in Scandinavia was 32.7 years and the sex distribution ratio of males to females was about 858: 429 [9]. The overall incidence of PCLR in the Italian population was 0.46 surgeries per 100,000 inhabitants per year, the median patient’s age was 30 years, and the male: female ratio was 5: 3 [10].

A better understanding of the epidemiological patterns of PCL injury and PCLR is vital in devising effective prevention and treatment strategies. A thorough epidemiological study will help not only to prevent injuries and diseases by enabling the identification of high-risk individuals based on known risk factors, but also to rebuild prevention programs targeting those high-risk individuals. A few studies have investigated epidemiological patterns of PCL injury and PCLR in Western countries [9–11]. However, to our knowledge, there has been no report on the epidemiological patterns of PCL injury and PCLR in the Asian population, including South Korea. As there are significant differences in social, cultural, and economic aspects between Asian and Western countries, the epidemiological patterns of PCL injury and PCLR in Asian countries would be distinct from those in Western countries. Therefore, understanding these patterns will be highly beneficial to understanding the unique characteristics of PCL injury and PCLR epidemiology in Asian populations and establishing proper public health care systems in Asian countries.

The aims of this study were as follows: (1) to determine the total number of cases per year and incidence per 100,000 people of PCLR through an analysis of nationwide insurance claim data in South Korea [the Health Insurance Review and Assessment Service (HIRA) database], (2) to determine the demographics of PCLR with respect to sex and age of patients between 2008 and 2016. With an increasing number of people engaging in higher levels of physical activities and older individuals staying physically active until later in their lives, it was hypothesized that there would be an increase in the incidence of PCLR during the study period.

## Methods

HIRA data are health insurance claims data, also called Korean National Health Insurance (NHI) data as it is generated in the process of reimbursing claims for healthcare services under the NHI system in South Korea. The HIRA data contains the medical billing data of the entire Korean population (97% health insurance and 3% medical care) [12, 13]. HIRA research data consists of six files: (1) the general information file; (2) the healthcare services file, including inpatient prescriptions; (3) the diagnoses file; (4) the outpatient prescriptions file; (5) the master drug file; and (6) the provider information file. The healthcare services file has specific and detailed information on healthcare services such as procedures, diagnostic tests, treatments, and inpatient prescriptions. In the diagnoses file, diagnoses are coded in compliance with Korean Standard Classification of Diseases Version 7 (KCD 7), which is based on the International Classification of Diseases, 10th Revision (ICD-10) [14].

The code of cruciate ligament reconstruction (N0880, N0881; Table 1) were not divided into anterior cruciate ligament reconstruction (ACLR) and PCLR; thus, patients who received PCLR was collected using the two following criteria; (1) patients with a cruciate ligament reconstruction (N0880, N0881), (2) patients with a diagnosis defined by KCD 7 codes for PCL injury (S8351, S8353, M2364; Table 1). The patients with a cruciate ligament repair procedure code (N0882) were excluded. After extracting PCLR data, the data was further analyzed as follows: (1) the total number of cases per year and incidence of PCLR per 100,000 (people/year), (2) epidemiological trend of PCLR between 2008 and 2016, (3) sex distribution of PCLR patients, and (4) age distribution of PCLR patients. Incidence per 100,000 people per year was based on additional information from the surveys, including basic epidemiologic data, as per the Korean National Statistics (KOSTAT). The study protocol was reviewed and approved by the Institutional Review Board (IRB) of our institute, and the IRB waived the requirements of informed consent as all data were anonymous. Therefore, this study was performed without prior informed consent.

**Table 1:** Surgical procedure codes and diagnostic codes of the HIRA

Surgical procedure codes for cruciate ligament reconstruction of the HIRA	
N0880	Simple cruciate ligament reconstruction
N0881	Complex cruciate ligament reconstruction
Diagnostic codes in the diagnoses file of the HIRA (KCD7 code)	
S8351	Sprain and strain of posterior cruciate ligament
S8353	Traumatic rupture of posterior cruciate ligament
M2364	Other spontaneous disruption of ligament(s) of knee, posterior cruciate ligament

Complex surgery including

a) remnant preserved reconstruction

b) reconstruction using additional accessory portals (including anteromedial or anterolateral)

c) double bundle reconstruction

d) revision reconstruction

*KCD 7* Korean Standard Classification of Diseases Version 7, *HIRA* The Health Insurance Review and Assessment Service

## Results

**Fig. 1:**
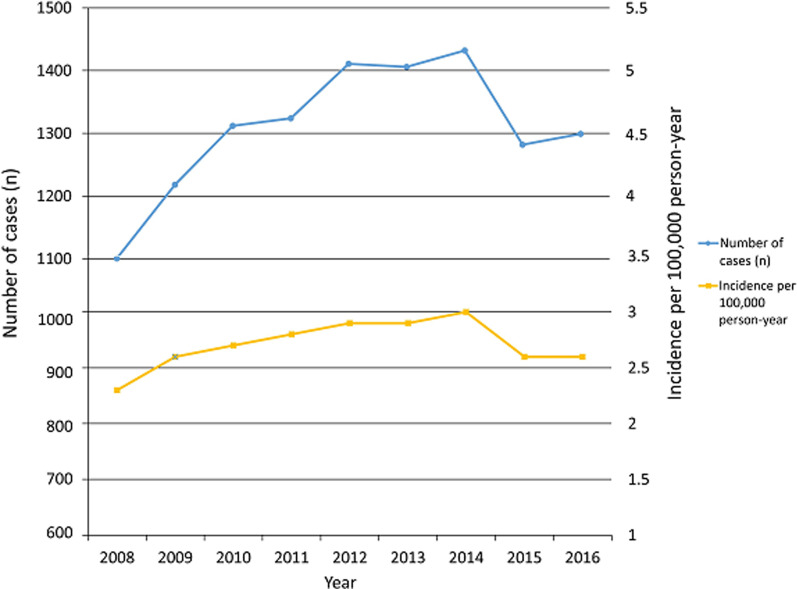
Total number and incidence per 100,000 people/year of posterior cruciate ligament reconstruction patients

The epidemiologic data and trends of PCLR between 2008 and 2016 in South Korea are presented in Table 2. The total number of PCLRs was 1101 in 2008 and increased to 1299 in 2016, and the incidence per 100,000 people/year was 2.3 in 2008 and increased to 2.6 in 2016 (Fig. 1). There was a 13% increase in the incidence of PCLR procedures over these 9 years.

**Table 2:** Epidemiologic data and trends in posterior cruciate ligament reconstruction in Korean population

Year	2008	2009	2010	2011	2012	2013	2014	2015	2016
Total cases	Case (*N*)	Case (*N*)	Case (*N*)	Case (*N*)	Case (*N*)	Case (*N*)	Case (*N*)	Case (*N*)	Case (*N*)
	1101	1218	1312	1324	1411	1406	1432	1282	1299
Sex									
Male	901	987	1049	1075	1108	1091	1125	1007	1000
Female	200	231	263	249	303	315	307	275	299
Age (years)									
< 20	131	146	156	140	146	174	177	147	137
20–29	304	327	352	307	289	308	325	312	275
30–39	291	312	330	261	235	211	232	185	194
40–49	252	278	295	297	302	280	302	278	288
≥ 50	123	155	179	319	439	432	396	360	405

Year	2008	2009	2010	2011	2012	2013	2014	2015	2016
Incidence	Per 100,000 people/year	Per 100,000 people/year	Per 100,000 people/year	Per 100,000 people/year	Per 100,000 people/year	Per 100,000 people/year	Per 100,000 people/year	Per 100,000 people/year	Per 100,000 people/year
	2.3	2.6	2.7	2.8	2.9	2.9	3.0	2.6	2.6
Sex									
Male	3.8	4.2	4.4	4.5	4.6	4.6	4.7	4.1	4.0
Female	0.8	1.0	1.1	1.0	1.3	1.3	1.3	1.1	1.2
Age (years)									
< 20	1.1	1.2	1.4	1.3	1.3	1.6	1.6	1.5	1.4
20–29	4.2	4.5	5.3	4.7	4.4	4.7	4.9	4.9	4.3
30–39	3.5	3.8	4.2	3.3	3.0	2.7	3.0	2.5	2.7
40–49	3.1	3.5	3.6	3.6	3.7	3.4	3.7	3.3	3.4
≥ 50	1.1	1.4	1.3	2.2	3.1	3.0	2.8	2.1	2.3

**Fig. 2:**
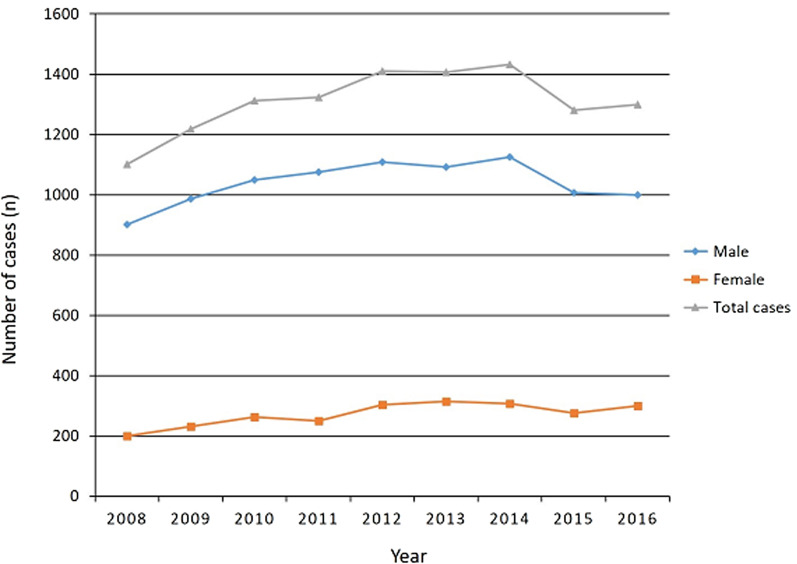
Total number of posterior cruciate ligament reconstruction patients per year stratified by sex

**Fig. 3:**
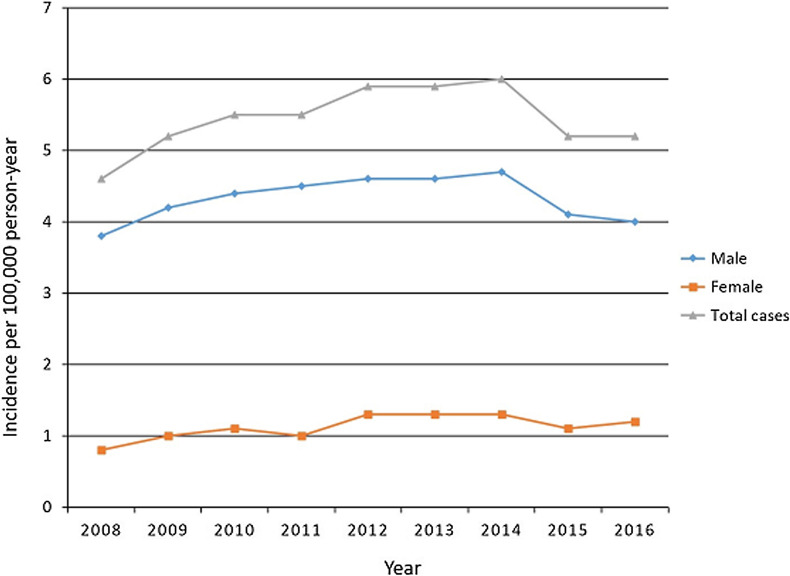
Incidence per 100,000 people/year of posterior cruciate ligament reconstruction patients stratified by sex

**Fig. 4:**
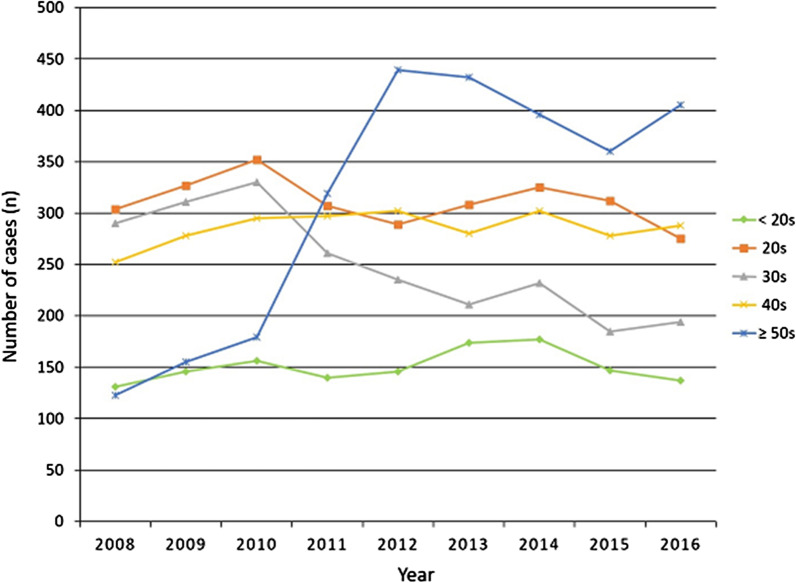
Total number of posterior cruciate ligament reconstruction patients per year stratified by age

**Fig. 5:**
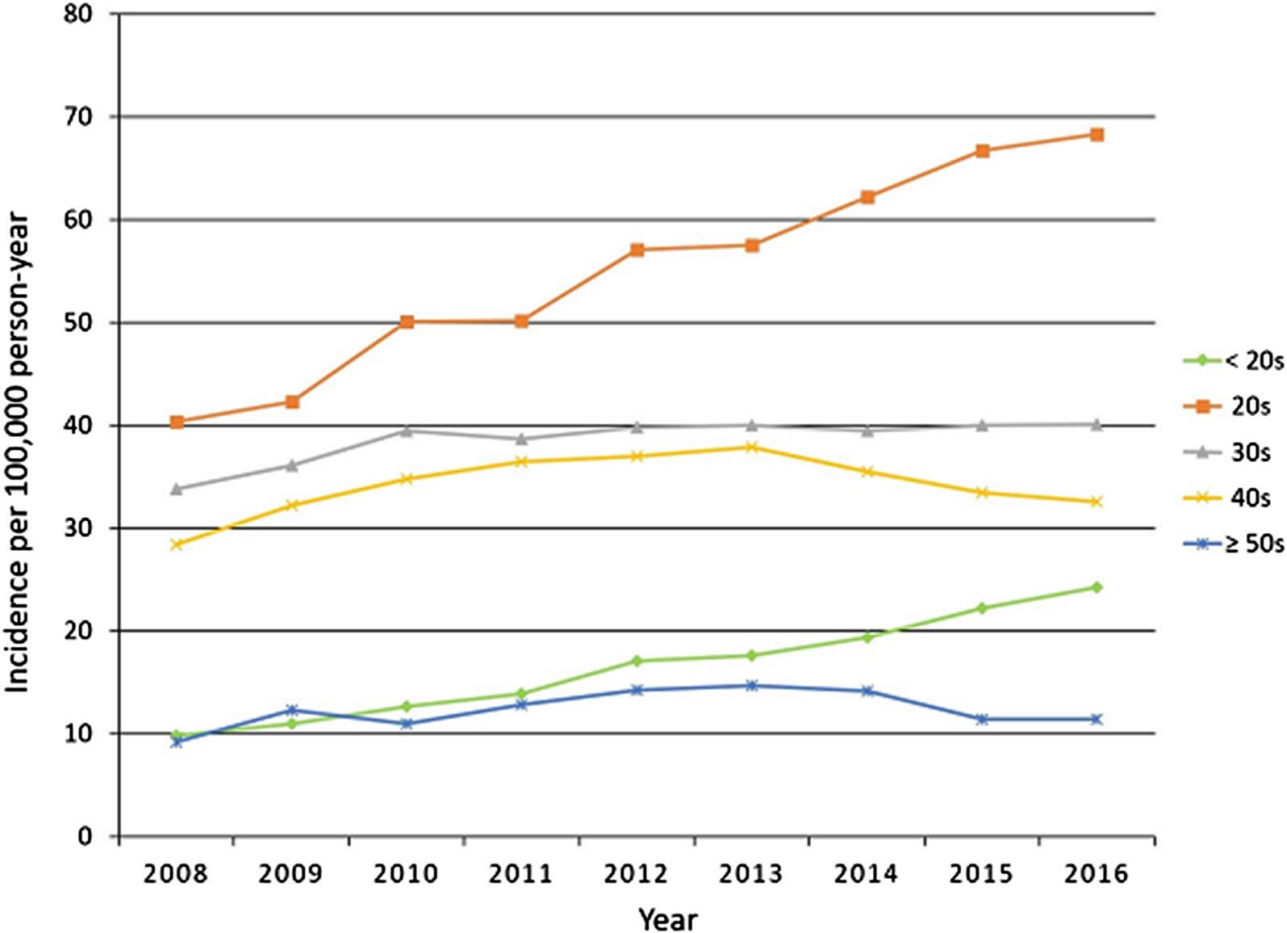
Incidence per 100,000 people/year of posterior cruciate ligament reconstruction patients stratified by age

Among males, the incidence was 3.8 (901 cases in total) in 2008 and significantly increased to 4.0 (1000 cases in total) in 2016 (Figs. 2 and 3). The incidence among females was 0.8 (200 cases in total) in 2016 and increased to 1.2 (299 cases in total) in 2016 (Figs. 2 and 3). Over this 9-year period, the incidence of PCLR in males and females increased by 5.2% and 50.0%, respectively. Although PCLR was performed more frequently in males, there was a higher increase in the incidence in females than males. The total number and incidence of PCLR between 2008 and 2016 in each age group is shown in Figs. 4 and 5, respectively. The highest incidence was seen in patients in their 20s, followed by those in their 40s and 30s.

## Discussion

The main aims of this study were to investigate the overall incidence and recent epidemiological trend of PCLR in South Korea. The important findings of this study are as follows: (1) the total incidence per 100,000 people and number of cases of PCLR procedures increased by 13% between 2008 and 2016, from 2.3 (1101 cases in total) to 2.6 (1299 cases in total); (2) a higher increase in the incidence of PCLR was seen in females compared with males, although PCLR was performed more frequently in males; (3) PCLR was performed most frequently in patients in their 20s, followed by patients in their 40s and 30s. The results of this study are consistent with the initial hypothesis that there will be an increase in the incidence of PCLR between 2008 and 2016, reflecting increased physical activity in children and the older population in recent years.

In South Korea, there is a legal obligation to include patient medical records in the HIRA database. Hence, insurance claim codes for surgical procedures in South Korea are prospectively recorded in the HIRA data. The authors obtained and analyzed data associated with the claim codes for cruciate ligament reconstruction from this nationwide database. Furthermore, the number of PCLR procedures was standardized as incidence per 100,000 people/year, for comparison with other countries. This study on the nationwide population is the first epidemiologic report of PCLR in Asian populations based on a nationwide database.

Prior to our study, there have been studies on the epidemiology of PCL injuries, using different data sources and collection methods, to investigate the overall effects of PCL injury and its consequences after reconstruction [9–11]. The overall incidence of PCLR in the Italian population was 0.46 surgeries per 100,000 inhabitants per year, the median patient age was 30 years, and the male:female ratio was 5: 3 [10]. Owesen et al. reported the epidemiology of 1287 patients who underwent PCLR from 2004 to 2013 in Scandinavian countries from the national ligament registries. They found that the average age of the treated patients was 32.7 years and the sex distribution ratio of male to female was 858:429 (66.7%: 33.3%) showing male patients are twice as likely to sustain PCL injury than female patients [9]. Schulz et al. also reported the epidemiology of 494 patients who underwent PCLR between 1993 and 1999 in their institution. The mean age at the time of injury was 27.5 years old, and 79.4% were male patients [11]. However, these studies did not investigate the total number of PCLR using nationwide standardized data such as the incidence per 100,000 (people/year).

The current study showed that the incidence of PCLR procedures increased by 13% over 9 years in South Korea. There are several possible reasons for an increase in PCLR during the study period. Higher engagement in physical and sports activities will lead to a greater chance of PCL injury [15]: the number of people engaging in high-level athletics can lead to an increase in PCLR. Due to improvements in surgical techniques, the operative approach has become increasingly popular [16], especially among people engaging in high-level athletics because of the possibility of a return to activity.

Patients in their 20s had the highest incidence of PCLR in South Korea, 4.3 per 100,000 people/year in 2016, followed by patients in their 40s and 30s. Interestingly, patients aged between 10 and 20 years had a lower incidence than patients in their 20s, 30s, and 40s in South Korea. Students in Western countries are encouraged to participate in sports activities during classes and after school sports activities, and their engagement in those activities during high school is considered as an important attribute in the college admission process [17]. However, sports activities in middle and high schools in South Korea have dropped dramatically as grades in physical education are not considered an important attribute in the notoriously competitive college admission process [18]. This difference in the age distribution of people engaged in a high level of physical activities between the Korean and Western populations would explain the highest incidence of PCLR in patients in their 20s in South Korea. The present study found that PCLR was performed frequently in patients in their 40s. A potential explanation for this would be clinically missed or delayed diagnosis of PCL ruptures, which has been missed in the acute phase and was first diagnosed later in the chronic phase [19, 20].

With respect to sex differences, PCLR procedures were performed approximately three times more frequently among males than females. It can be presumed that males are more likely to engage in high-level athletics or high-risk activities than females. However, the increase rate was higher in females (50.0%) than males (5.2%) over these 9 years. This could be due to an increase in women’s engagement in sporting activities in South Korea during this period. In Scandinavian countries, female and male patients accounted for 33.3% and 66.7%, respectively, of the PCL injuries performed between 1993 and 1999 [9]. The present study demonstrated a relatively lower incidence of PCLR in females in South Korea compared with Scandinavian countries, although the rate of PCLR steadily increased in South Korea. Traditionally, interest and participation in sports and other physical activities among females in South Korea has been lower than those in Western countries. However, women’s engagement in sports in South Korea has recently increased, and this may explain the recent increase of PCLR among Korean women [17, 18, 21, 22].

The importance of our study lies in the fact that this is an uncommon study investigating the nationwide trends of PCLR based on a national database in Asian countries, whose social and cultural backgrounds and health care systems are different from those of Western countries. This national database is one of the most comprehensive and accurate national databases in the world as it covers the entire Korean population, including all PCLR records, and is managed by the Korean government.

The present study is clinically relevant as a higher rate of engagement in physical and sports activities will lead to a higher chance of PCL injury in the population, especially people in their 20s and females. This will contribute to understanding the epidemiology of PCLR in South Korea, and will aid in the development of national healthcare policies and cost-effective preventive programs with regard to demographics including sex and age.

However, several limitations exist in the current study. First, in this study, only PCLR data was reported, and PCL injury data was not investigated since the initial research focus was PCLR, and extracting specific PCL injury data was limited. It is not possible to know whether the increase in the number of PCL surgeries was due to an increase in PCL injury incidence or surgical treatment rate. It is important to further investigate epidemiological data of PCL injuries in future studies. In addition, the current study did not include PCL repair data. However, the total number of PCL repair cases may be much smaller than that of PCLR in the HIRA database. Second, HIRA data was collected for administrative purposes, not for research; therefore, many important data attributes related to this PCL investigation (such as limb laterality, acute or chronic, mechanism of injuries, combined injuries, and specific reasons for injuries) were not available. However, this issue was not only found in HIRA data, but also in other claims databases. Third, the PCLR cases in our analysis did not include occupational injury cases; however, the total number of occupational injury cases is much smaller than those recorded in the HIRA database. Fourth, Korean national insurance can cover traffic accident injuries, thus HIRA data includes traffic accident data. However, since traffic accident data cannot be classified by code, the percentage of traffic accident injuries could not be investigated in this study. Fifth, primary, revision, and bilateral reconstruction cases were included in our analysis as these were recorded under the same claim code in the HIRA database. Sixth, this dataset contained only sex and age. The data was insufficient for establishing a prevention strategy and healthcare system. More data, such as social status, occupation, cause of injury, insurance, etc., are required to identify the factors that cause PCL and establish risk prevention.

## Conclusion

The incidence of PCLR increased by 13% over 9 years in South Korea. PCLR was performed approximately three times more in men than women. The incidence of PCLR was highest in people in their 20s, followed by those in their 40s. The current study will enhance our understanding of the epidemiology of PCLR.

## Data Availability

The datasets developed during and/or analyzed during the current study are available from the corresponding author on reasonable request.

## Abbreviations

ACLR: Anterior cruciate ligament reconstruction
HIRA: Korean Health Insurance Review and Assessment Service
ICD-10: The International Classification of Diseases 10th Revision
IRB: Institutional Review Board
KCD 7: Korean Standard Classification of Diseases Version 7
KOSTAT: Korean National Statistics
NHI: Korean National Health Insurance
PCL: Posterior cruciate ligament
PCLR: Posterior cruciate ligament reconstruction

## References

[1] Jacobi M, Reischl N, Wahl P, Gautier E, Jakob R (2010). Acute isolated injury of the posterior cruciate ligament treated by a dynamic anterior drawer brace: a preliminary report. J Bone Joint Surg Br.

[2] Patel DV, Allen AA, Warren RF, Wickiewicz TL, Simonian PT (2007). The nonoperative treatment of acute, isolated (partial or complete) posterior cruciate ligament-deficient knees: an intermediate-term follow-up study. HSS J.

[3] LaPrade CM, Civitarese DM, Rasmussen MT, LaPrade RF (2015). Emerging updates on the posterior cruciate ligament: a review of the current literature. Am J Sports Med.

[4] Patel DV, Allen AA, Warren RF, Wickiewicz TL, Simonian PT (2007). The nonoperative treatment of acute, isolated (partial or complete) posterior cruciate ligament-deficient knees: an intermediate-term follow-up study. HSS J.

[5] Shelbourne KD, Clark M, Gray T (2013). Minimum 10-year follow-up of patients after an acute, isolated posterior cruciate ligament injury treated nonoperatively. Am J Sports Med.

[6] Boynton MD, Tietjens BR (1996). Long-term followup of the untreated isolated posterior cruciate ligament-deficient knee. Am J Sports Med.

[7] Wang SH, Chien WC, Chung CH, Wang YC, Lin LC, Pan RY (2018). Long-term results of posterior cruciate ligament tear with or without reconstruction: a nationwide, population-based cohort study. PLoS ONE.

[8] Lind M, Nielsen TG, Behrndtz K (2018). Both isolated and multi-ligament posterior cruciate ligament reconstruction results in improved subjective outcome: results from the Danish Knee Ligament Reconstruction Registry. Knee Surg Sports Traumatol Arthrosc.

[9] Owesen C, Sandven-Thrane S, Lind M, Forssblad M, Granan LP, Aroen A (2017). Epidemiology of surgically treated posterior cruciate ligament injuries in Scandinavia. Knee Surg Sports Traumatol Arthrosc.

[10] Longo UG, Viganò M, Candela V, De Girolamo L, Cella E, Thiebat G, Salvatore G, Ciccozzi M, Denaro V (2021). Epidemiology of posterior cruciate ligament reconstructions in Italy: a 15-year study. J Clin Med.

[11] Schulz MS, Russe K, Weiler A, Eichhorn HJ, Strobel MJ (2003). Epidemiology of posterior cruciate ligament injuries. Arch Orthop Trauma Surg.

[12] Song S, Lee SE, Oh SK, Jeon SA, Sung JM, Park JH, Chang HJ (2018). Demographics, treatment trends, and survival rate in incident pulmonary artery hypertension in Korea: a nationwide study based on the health insurance review and assessment service database. PLoS ONE.

[13] Chung KS, Ha JK, Kim YS, Kim JH, Ra HJ, Kong DH, Wang PW, Choi CH, Kim JG (2019). National trends of meniscectomy and meniscus repair in Korea. J Korean Med Sci.

[14] Kim J-A, Yoon S, Kim L-Y, Kim D-S (2017). Towards actualizing the value potential of Korea health insurance review and assessment (HIRA) data as a resource for health research: strengths, limitations, applications, and strategies for optimal use of HIRA data. J Korean Med Sci.

[15] Krutsch W, Zeman F, Zellner J, Pfeifer C, Nerlich M, Angele P (2016). Increase in ACL and PCL injuries after implementation of a new professional football league. Knee Surg Sports Traumatol Arthrosc.

[16] Fanelli GC (2018). Knee dislocation and multiple ligament injuries of the knee. Sports Med Arthrosc Rev.

[17] Fox CK, Barr-Anderson D, Neumark-Sztainer D, Wall M (2010). Physical activity and sports team participation: associations with academic outcomes in middle school and high school students. J Sch Health.

[18] Cho M, Kwon W-D, Jeon Y-B (2010). Are Korean secondary school girls physically active during leisure time?. Health Care Women Int.

[19] Andrews JR, Edwards JC, Satterwhite YE (1994). Isolated posterior cruciate ligament injuries. History, mechanism of injury, physical findings, and ancillary tests. Clin Sports Med.

[20] Shelbourne KD, Rubinstein RA (1994). Methodist Sports Medicine Center's experience with acute and chronic isolated posterior cruciate ligament injuries. Clin Sports Med.

[21] Choi JY, Chang AK, Choi EJ (2015). Sex differences in social cognitive factors and physical activity in Korean college students. J Phys Ther Sci.

[22] Sood M, Kulshrestha V, Sachdeva J, Ghai A, Sud A, Singh S (2020). Poor functional outcome in patients with voluntary knee instability after anterior cruciate ligament reconstruction. Clin Orthop Surg.

